# A review on the current status and definitions of activity indices in inflammatory bowel disease: how to use indices for precise evaluation

**DOI:** 10.1007/s00535-022-01862-y

**Published:** 2022-03-02

**Authors:** Masahiro Kishi, Fumihito Hirai, Noritaka Takatsu, Takashi Hisabe, Yasumichi Takada, Tsuyoshi Beppu, Ken Takeuchi, Makoto Naganuma, Kazuo Ohtsuka, Kenji Watanabe, Takayuki Matsumoto, Motohiro Esaki, Kazutaka Koganei, Akira Sugita, Keisuke Hata, Kitarou Futami, Yoichi Ajioka, Hiroshi Tanabe, Akinori Iwashita, Hirotaka Shimizu, Katsuhiro Arai, Yasuo Suzuki, Tadakazu Hisamatsu

**Affiliations:** 1 Inflammatory Bowel Disease Center, Fukuoka University Chikushi Hospital, Chikushino, Japan; 2 Department of Gastroenterology, Fukuoka University Faculty of Medicine, 7-45-1 Nanakuma, Jonan-ku, Fukuoka City, Fukuoka 814-0180 Japan; 3 Department of Gastroenterology, Fukuoka University Chikushi Hospital, Chikushino, Japan; 4 Tsujinaka Hospital Kashiwanoha, Kashiwa, Japan; 5 The Third Department of Internal Medicine, Kansai Medical University, Osaka, Japan; 6 Department of Endoscopy, Tokyo Medical and Dental University, Tokyo, Japan; 7 Center for Inflammatory Bowel Disease, Division of Internal Medicine, Hyogo College of Medicine, Hyogo, Japan; 8 Division of Gastroenterology, Department of Medicine, Iwate Medical University, Iwate, Japan; 9 Division of Gastroenterology, Department of Internal Medicine, Faculty of Medicine, Saga University, Saga, Japan; 10 Department of Inflammatory Bowel Disease, Yokohama Municipal Citizen’s Hospital, Yokohama, Japan; 11 Nihonbashi Muromachi Mitsui Tower Midtown Clinic, Tokyo, Japan; 12 Department of Surgery, Fukuoka University Chikushi Hospital, Chikushino, Japan; 13 Division of Molecular and Diagnostic Pathology, Graduate School of Medicine and Dental Sciences, Niigata University, Niigata, Japan; 14 Department of Pathology, Fukuoka University Chikushi Hospital, Fukuoka, Japan; 15 AII Research Institute of Pathology and Image Diagnosis, Fukuoka, Japan; 16 Center for Pediatric Inflammatory Bowel Disease, Division of Gastroenterology, National Center for Child Health and Development, Tokyo, Japan; 17 Ginza Central Clinic, Tokyo, Japan; 18 Department of Gastroenterology and Hepatology, Kyorin University School of Medicine, Tokyo, Japan

**Keywords:** Inflammatory bowel disease, Ulcerative colitis, Crohn’s disease, Clinical index, Endoscopic index

## Abstract

Many clinical trials have been conducted for inflammatory bowel disease (IBD), so various clinical indices (CIs) and endoscopic indices (EIs) have also been evaluated. However, recently, with the progress of IBD management, review of established indices from previous studies, and establishment of new indices, the landscape of the use of indices in clinical trials have changed. We investigated the number and frequency of the indices adapted in recent clinical trials for ulcerative colitis (CI and EI) and Crohn’s disease (CI, EI, index related to magnetic resonance imaging, index for evaluating patient-reported outcomes, and health-related quality of life). Based on the results, we selected representative indices and further reviewed their content and characteristics. Moreover, various definitions, including clinical and endoscopic response or remission, have been described by means of representative indices in clinical trials.

**Supplementary Information:**

The online version contains supplementary material available at 10.1007/s00535-022-01862-y.

## Introduction

Inflammatory bowel disease (IBD) is treated based on the activity and extent of the disease. Evaluations using the clinical index (CI) and endoscopic index (EI) are useful in determining the appropriate treatment, predicting prognosis, and evaluating or monitoring disease activity after treatment [1, 2]. Generally, clear treatment evaluation is important while performing clinical trials for IBD. However, treatment goals have diversified into clinical, endoscopic, pathological, and psychological goals recently [1–3]. Additionally, the indices used in recent clinical trials have changed due to the review of previously established indices, establishment of new indices, and diversification of treatment goals, such as mucosal healing (MH) and pathological healing (PH). In response to this background, the Selecting Therapeutic Target in IBD (STRIDE) program was established in 2013 by the International Organization for the Study of IBD. An achievement of this program, STRIDE-I was presented in 2015 as a recommended treat-to-target (T2T) approach based on the evidence and consensus of experts [4]. In 2020, a more updated STRIDE-II was presented [5]. Practicing T2T is important for long-term treatment strategies for IBD [4–6], and STRIDE-II describes the importance of achieving treatment goals, which are suitable for early, middle, and long-term treatments [5]. The disease activity index based on clinical symptoms; biomarkers, such as C-reactive protein (CRP) or fecal calprotectin (FC), endoscopic index, and histological index, are widely used to monitor disease activity. In this review, we first clarified the indices adopted frequently in clinical trials for IBD, and derived representative indices for ulcerative colitis (UC) and Crohn’s disease (CD). We then outlined how clinical and endoscopic remissions were defined in these clinical trials provided that each index has problems with its characteristics and definitions. The purpose of this review was to optimize the method of evaluating disease activity in IBD and help select and define appropriate indicators when planning clinical trials.

## Ulcerative colitis

UC is a refractory, chronic, IBD involving the large intestine. Activity index requires accuracy and validity for selecting its treatment and evaluating its efficacy on a patient post treatment [3, 7–9]. The establishment of effective treatments, such as biologics, ensured the improvement of therapeutic goals, including endoscopic or histological remission, and the improvement of symptoms that had been defined as conventional goals. Therefore, as the evaluation items or the content required by an index used continue to change, the index adopted in clinical trials also changes. For example, the placebo effect is not suppressed by the evaluation of the CI comprising only subjective symptoms as their subjective evaluations have limitations [1, 7, 10, 11]. Therefore, most recent clinical trials for UC have adopted biomarker levels or endoscopic evaluation as the main parameters for objective evaluation. Clinical trials for UC frequently adopt the Mayo Score, which includes the CI-independent endoscopic score or the EI as a sub-score item. Considering these, to clarify the circumstances of adopted indices in recent clinical trials performed for UC, we conducted a comprehensive literature search through PubMed with a survey period spanning from January 2009 to December 2017. In 2010, Hirai et al. carefully read 100 papers with “ulcerative colitis” and “clinical trial” in the search string from 2001 to 2006 and reported the frequency of CIs and EIs used [12]. Thereafter, an additional survey was conducted using the same method during the search period of 1999–2008 as a framework of the Ministry of Health, Labour and Welfare of Japan. In creating this review, we followed the research method of Hirai et al. and investigated the indices used in studies in 2009–2017. To clarify the change of the index used over time, the research results were divided into three categories for each survey period: 1999–2008, 2009–2012, and 2013–2017.

**Fig. 1:**
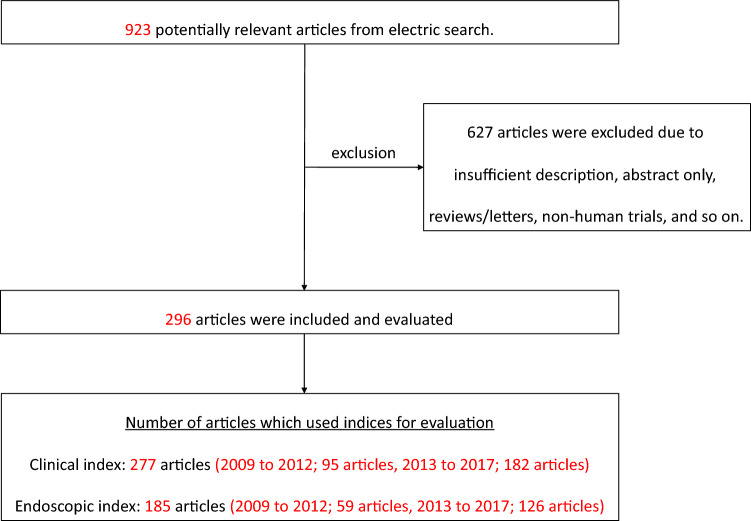
Flow diagram of the study selection process for UC. We performed an electronic search using PubMed. The survey period was from January 2009 to December 2017. The search string we used included “ulcerative colitis” and “clinical trial” as key words. We carefully read the studies that were extracted, and 296 studies were considered to have been evaluated by an index

With “ulcerative colitis” and “clinical trial” included in the search string, 296 articles regarded as evaluated with CI or EI were extracted and read through carefully. Figure 1 shows the flow diagram of the study selection process and exclusion criteria (e.g., insufficient descriptions, abstract only, general remarks, letters, and studies not targeting humans). Tables 1 and 2 show the frequency of CI and EI use based on these research results.

**Table 1:** Number and frequency of CIs in recent clinical trials for UC

Survey period	Index	Validation	Number (%)
1999–2008 (*N* = 215, including duplicates)			
	CAI score (Including Rachmilewitz index)	Validated	58 (37.0%)
	DAI score (Including Sutherland index)	Not validated	38 (17.7%)
	Mayo score (Including partial Mayo Score)	Not validated	25 (11.6%)
	Lichtiger index	Not validated	21 (9.8%)
	Truelove and Witts index	Not validated	17 (7.9%)
	Powel–Tuck index (St. Mark’s index)	Not validated	8 (3.7%)
	Simple Clinical Colitis Activity Index (SCCAI)	Validated	8 (3.7%)
	Seo index	Validated	6 (2.8%)
	Others (Including original indices for the trial)	Not available	36 (16.7%)
2009–2012 (*N* = 95, including duplicates)			
	DAI score (Including Sutherland Index)	Not validated	29 (30.5%)
	Mayo score (Including partial Mayo Score)	Not validated	26 (27.4%)
	CAI score (Including Rachmilewitz Index)	Validated	19 (20.0%)
	Simple Clinical Colitis Activity Index (SCCAI)	Validated	7 (7.4%)
	Lichtiger index	Not validated	6 (6.3%)
	Truelove and Witts index	Not validated	4 (4.2%)
	Pediatric Ulcerative Colitis Activity Index (PUCAI)	Validated	3 (3.2%)
	Pouchitis Disease Activity Index (PDAI)	Not validated	2 (2.1%)
	Others (Including original indices for the trial)	Not available	5 (5.3%)
2013–2017 (*N* = 182, including duplicates)			
	Mayo score (Including partial Mayo Score)	Not validated	90 (49.5%)
	CAI score (Including Rachmilewitz Index)	Validated	30 (16.5%)
	DAI score (Including Sutherland Index)	Not validated	25 (13.7%)
	Simple Clinical Colitis Activity Index (SCCAI)	Validated	21 (11.5%)
	Pediatric Ulcerative Colitis Activity Index (PUCAI)	Validated	21 (11.5%)
	Pouchitis Disease Activity Index (PDAI)	Not validated	6 (3.3%)
	Lichtiger index	Not validated	4 (2.2%)
	Powell–Tuck index (St. Mark’s index)	Not validated	2 (1.1%)
	Others (Including original indices for the trial)	Not available	5 (2.7%)

*CAI* clinical activity index, *CIs* clinical indices, *DAI* disease activity index, *UC* ulcerative colitis

**Table 2:** Number and frequency of EIs in recent clinical trials for UC

Survey period	Index	Validation	Number (%)
1999–2008 (*N* = 147, including duplicates)			
	Baron score	Validated	68 (46.2%)
	Rachmilewitz endoscopic index	Not validated	35 (23.8%)
	Matts score	Not validated	6 (4.1%)
	Tygat Endoscopic grading score	Unknown	4 (2.7%)
	Others (Including original indices for the trial)	Not available	34 (23.1%)
2009–2012 (*N* = 58, including duplicates)			
	Mayo Score (Including Mayo Endoscopic Sub-score)	Not validated	25 (42.4%)
	DAI Score (Including Sutherland Index)	Not validated	24 (40.7%)
	Rachmilewitz endoscopic index	Not validated	5 (8.5%)
	Pouchitis Disease Activity Index (PDAI)	Not validated	2 (3.4%)
	Matts Classification	Not validated	2 (3.4%)
	Others (Including original indices for the trial)	Not available	5 (8.5%)
2013–2017 (*N* = 126, including duplicates)			
	Mayo Score (Including Mayo Endoscopic Sub-score)	Not validated	87 (69.0%)
	DAI Score (Including Sutherland Index)	Not validated	25 (19.8%)
	Baron Score (Including modified Baron Score)	Validated	7 (5.6%)
	Pouchitis Disease Activity Index (PDAI)	Not validated	6 (4.8%)
	Rachmilewitz endoscopic index	Not validated	6 (4.8%)
	Ulcerative Colitis Endoscopic Index of Severity (UCEIS)	Validated	6 (4.8%)
	Matts Classification	Not available	5 (4.0%)

*DAI* disease activity index, *EIs* endoscopic indices, *UC* ulcerative colitis

The indices revealed to be used most frequently in previous studies, considered to be highly valid, and appeared to be frequently applicable in future were selected, and their respective development reasons, evaluation items, characteristics, and various definitions were reviewed. Additionally, the histopathological, pediatric UC, and postoperative ileal pouchitis indices were described in sections independent from the results of the research.

### Results of electronic research for UC

In terms of most recent (2013–2017) CIs for adults, Mayo Score (49.5%), Rachmilewitz Index (also known as Clinical Activity Index, CAI) (16.5%), Sutherland Index (also known as Disease Activity Index, DAI) (13.7%), Simple Clinical Colitis Activity Index (SCCAI) (11.5%), and Lichtiger Index (2.2%) have been most adopted.

In terms of most recent (2013 to 2017) EIs, Mayo Endoscopic Sub-score (MES) (69.0%) and Sutherland Endoscopic Sub-score (19.8%), regarded as a sister index for MES, accounted for an overwhelmingly large percentage. They were followed by the Baron index (5.6%), Rachmilewitz endoscopic sub-score (4.8%), and Ulcerative Colitis Endoscopic Index of Severity (UCEIS) (4.8%).

The survey results clarified that the usage rate of Mayo score (including partial Mayo score) has been increasing in recent CIs. It also clarified that the usage rate of Mayo score (including Mayo endoscopic sub-score) has been increasing for recent EIs. In addition, the recently developed Ulcerative Colitis Endoscopic Index of Severity (UCEIS) was used for the evaluations in some recent studies.

Among CIs, the Mayo, Sutherland, Rachmilewitz, Simple Clinical Colitis Activity, and Lichtiger indices were described. For the EIs, the Sutherland endoscopic sub-score is regarded as nearly similar to MES, so only MES was selected. Along with UCEIS, whose validity has been evaluated, MES was described based on the results of adoption frequency. Additionally, the histopathological, pediatric UC, and postoperative ileal pouchitis indices were described in sections independent from the results of the research.

### Clinical Index for UC

#### Mayo Score (Supplementary Table 1)

Schroeder et al. developed this index to evaluate the efficacy and safety of oral mesalazine [13]. Eighty-seven patients with mild to moderate active UC were administered mesalazine and placebo for six weeks, and evaluated in a randomized, double-blind, controlled trial. In the original article, the efficacy was evaluated by comparing the proportions of complete response (CR), partial response (PR), and no response in each administration group with those in the placebo group. The Mayo Score has since then been the most adapted index in recent clinical trials [5]. However, an index that is an independently modified Mayo Score is also frequently used. Since then, the Mayo Score has been frequently used as a partial Mayo Score (p-MS) or as a MES, combining one or multiple items of the sub-score. Although the Mayo Score is simple and frequently used, its validity has yet to be examined sufficiently, and its definitions of CR and MH have remained inconsistent. The MES is described independently in the section on EI.

#### Sutherland Index (Supplementary Table 2)

Sutherland et al. developed this index to easily evaluate the efficacy and safety of mesalazine enemas [14]. Endoscopic mucosal findings, which are quoted from the Baron Index, are applied to evaluate the activity. It is possible not only to evaluate the index as a whole, but also evaluate each evaluation item independently. Although it is composed of almost the same items as the Mayo Score, each evaluation item, except for those related to endoscopic findings, is evaluated based on the condition of a patient after 1 day, which is different from the 3-day period of the Mayo Score.

#### Rachmilewitz Index (Supplementary Table 3)

Rachmilewitz et al. developed this index to compare the efficacy and safety of mesalazine and sulfasalazine [15]. CAI and EI were proposed separately. CAI is evaluated based on the condition of a patient 1 week before further evaluation. EI scores each of the four characteristic findings of UC during its active period and uses their sum as the score. There is a discrepancy between the remission rates of CAI and EI, and CAI and EI do not necessarily correlate. Additionally, CAI has been validated, but EI has not.

#### Simple Clinical Colitis Activity Index (Supplementary Table 4)

Walmsley et al. developed this index to evaluate disease activity easily [16]. Based on the Powell-Tuck Index, bowel frequency (night) was included in the nighttime symptoms evaluated. In the original article, its evaluation items are described simply enough for non-specialists or even patients themselves to perform during initial evaluations at the outpatient department. Additionally, its high correlation with the scoring system of the Seo Index, which is a well-validated index including three objective contents (hemoglobin, erythrocyte segmentation ratio, and albumin), has been confirmed [16, 17].

#### Lichtiger Index (Supplementary Table 5)

Lichtiger et al. developed this index to evaluate the efficacy and safety of continuous intravenous cyclosporine therapy [18, 19]. The purpose of this index is to evaluate severe patients, and since the evaluation period for them is short, it focuses on short-term changes in symptoms. Therefore, it requires a condition in which the evaluation of efficacy is continuous for 2 consecutive days. Additionally, the reliability of this index is suggested by the fact that there was no difference in the evaluation of two or more physicians and that the improvement rate under blinding and after key opening were similar.

### Endoscopic Index for UC

#### Mayo endoscopic sub-score

Among the evaluation items included in the Mayo Score, only items related to mucosal findings of the endoscopy were used independently as EI, which is called MES. Although it is just a simple, four-grade evaluation, it still has the following problems: the score may not change much before and after treatment and does not reflect partial or minor changes in vascular visibility.

##### Issues of evaluation of mucosal healing using MES

While the MES is simple and frequently used, its validity has yet to be sufficiently examined, and its definitions of CR and MH have remained inconsistent. A frequently used definition of MH is “MES = 0” or “MES = 1” [5, 20, 21]. However, there is a difference in the relapse rate between “MES = 0” and “MES = “1 [20–25]. Therefore, the achievement of MES 0 is recommended for better outcomes in the recent literature composed of voting results by IBD experts [5]. Additionally, the MES scores appear to vary depending on the evaluation site or physician.

#### Ulcerative Colitis Endoscopic Index of Severity (Supplementary Table 6)

Regarding EIs used before this index was established, differences in evaluation between physicians were pointed out, and none of them were validated. Travis et al. pointed out these problems, judged various endoscopic findings in UC reported by multiple physicians, verified their validity sufficiently, and identified three findings that are optimal for endoscopic activity evaluation: “Vascular pattern”, “Bleeding”, and “Erosions and ulcers”. A highly objective index was then developed in which these three findings were evaluated and scored separately at the site with the strongest findings prior to calculating for the total score [26, 27]. This index makes it possible to easily evaluate changes in each finding and to evaluate severity more objectively compared to the grading scale represented by MES. It also has the advantage of having a wide score range, 0–8 points, making the degree of improvement before and after treatment easier to evaluate objectively.

##### Issues of evaluation of mucosal healing using UCEIS

Although “endoscopic remission (ER)” or “MH” is not defined in the original article, there is a high consensus that it refers to “UCEIS = 0” or “UCEIS ≤ 1” [5, 21]. However, knowledge about the long-term prognosis and surgical rate of UC based on UCEIS is limited at present and needs further investigations [28–30].

##### Reproducibility between MES and UCEIS

A good correlation between MES and UCEIS was suggested (κ = 0.713, *p* < 0.001) [32]. In addition, when the weighted kappa index in MES was 0.8 (good), 0.52 (acceptable), and 0.49 (acceptable) in the evaluation of the same case by three endoscopists, the intra-class correlation coefficient of UCEIS was 0.92 (95% CI 0.83–0.96), indicating a correlation between MES and UCEIS [33]. On the other hand, since the degree of ulceration (shallow or deep) was not mentioned in the MES, it may lead to a discrepancy between MES and UCEIS [28].

### Histopathological index for UC

The evaluation of UC activity has been based primarily on clinical symptoms and endoscopic findings [1]. However, the importance of histological improvement has recently been highlighted based on various histopathological indices [31, 34]. Conversely, the correlations between ER and histopathological remission (HR) and inter-observer reliability in the evaluation of histopathological inflammation are relatively weak [35, 36]. Therefore, the clinical utility of STRIDE-II on histological improvement is limited; in fact, it was deemed low by the Delphi method, positioning this problem as a gap that needs to be addressed by future investigations [5]. Currently, the achievement of HR is rarely set as the primary endpoint of UC treatment, but it is recommended that histopathological evaluation be set as a secondary endpoint [8]. To better illustrate this, this section describes the Matts, Riley, and Geboes scores.

#### Matts Classification (Supplementary Table 7)

Matts et al. developed this index for endoscopic and histopathological evaluation of UC in a review of rectal biopsy procedures and reported a simple and safe method [37]. Histological grades of the Matts classification are classified into five grades (1–5) based on histopathological findings (cellular infiltration, crypt abscesses, erosion, and ulcer).

#### Riley Score (Supplementary Table 8)

Riley et al. developed this index to evaluate relapse based on microscopic rectal inflammation [38]. It is a grading scale that evaluates six histopathological findings on a 4-point scale with the average of the evaluations made by the two pathologists considered as the final score. “Inflammatory cell infiltrate”, “crypt abscesses”, “mucin depletion”, and “breaches in the surface epithelium” showed a high correlation with relapse. It is important to note that this index is different from the grading systems developed by Riley et al. in 1988 [39]. Although a clear definition of remission is not shown in the original article, definitions, such as “Riley Score = 1” [40] and “Riley Score change ≥ Δ1” [40, 41], are applied as “remission” and “improvement”, respectively.

#### Geboes Score (Supplementary Table 9)

Indices for histopathological evaluation focusing on neutrophil infiltration have been proposed, but their validity has yet to be verified. Therefore, Geboes et al. developed this index to achieve clear validity [42]. The overall evaluation concordance rate was 65%, which was considered a “good or acceptable quality”. The main advantage of this index is that each evaluation item is evaluated independently. Unlike conventional indices, evaluation of activity is performed not only by one item, such as degree of neutrophil infiltration, but also by other evaluation items. Although the definition of remission is not clearly stated in the original article, patients with “Geboes Score ≤ 1.0” [43] and “Geboes Score < 2.0” [44] are considered in “remission”.

### Index for Pediatric UC

Evaluating pediatric UC patients using the results of laboratory findings or imaging findings, including endoscopy, has proven to be more difficult than evaluating adult UC patients. Additionally, some indices that have been developed independently focus only on subjective symptoms. The earlier onset of UC is characterized by a higher tendency to become severe and develop into total colitis types [45, 46]. Unlike in adults, a higher dose of medication is recommended, pointing out the growing trend of dependence on steroids and immuno-modulators [47]. This section describes the Pediatric UC Activity Index (PUCAI), which is an index for pediatric UC that is regarded as important in terms of treatment selection.

#### Pediatric Ulcerative Colitis Activity Index (Supplementary Table 10)

Turner et al. developed this index to evaluate disease activity in pediatric UC cases with a noninvasive and accurate reflection [48]. Item reduction and instrument formatting were carried out using the Delphi technique, and the items were weighted using Physician’s Global Assessment (PGA). Good correlation was confirmed with PGA, Total Colonoscopy Activity Index and Mayo Score. Additionally, its inter-observer reliability (ICC > 0.87) and test–retest reliability (ICC = 0.94) were also good. Subsequent studies reported that PUCAI on days 3 and 5 of steroid therapy was useful in assessing steroid responsiveness and in deciding to move on to the next treatment [49, 50]. In STRIDE-II, this index is recommended as a CI for pediatric UC [5].

### Index for postoperative ileal pouchitis

Although the surgery rate of UC has decreased due to advances in medical treatment, surgery is still needed for intractable cases with severe bleeding, perforation, addictive megacolon, and patients with colitis-associated dysplasia or cancer [51–57]. Postoperative complications such as ileal pouchitis are observed with a certain probability, and in some cases, it is difficult to control [56]. Objective evaluation of ileal pouchitis is therefore very important in determining the most appropriate management strategies and evaluating subsequent treatments. This section describes the Pouchitis Disease Activity Index (PDAI), which is an index of ileal pouchitis.

#### Pouchitis Disease Activity Index (Supplementary Table 11)

Sandborn et al. developed the PDAI to evaluate ileal pouchitis after ileal pouch-anal anastomosis, and compared it with previously reported indices for ileal pouchitis (Pouchitis Triad, Histopathology Index) [57]. PDAI showed significantly higher scores in patients who had clinical symptoms of ileal pouchitis than in other patients. The definition of pouchitis activity was not referred to in the original article.

## Crohn’s disease

CD presents with complications, such as stenosis, fistula, and abscess, and approximately 50% of cases will undergo surgery within 10 years of diagnosis [54, 58]. Additionally, its risks for postoperative relapse, mainly at the anastomotic site, and complicating to colorectal cancer have been reported [54, 58, 59]. In such disabilities, the progression of disease behavior over time remains to be a problem. In recent years, various effective treatments, including biologics, have become generalized, and treatment targets such as “MH” and “deep remission” have become widespread. In addition, as with UC, the concept of a T2T approach that makes use of biomarker normalization to achieve the targeted ER has been presented [1, 2, 4, 5, 60]. However, there remains no consensus on the clear definitions of clinical and endoscopic responsiveness and remission, which are the goals of monitoring [60, 61]. In STRIDE-II, treatment targets are set for each period, and strategies that are more in line with actual clinical practice are recommended [5]. In future CD treatment, monitoring with biomarkers and endoscopy will be important, but it seems important to first clarify the circumstances of more recent clinical trials related to CD that made them adopt specific indices. In the same manner the UC indices were analyzed, these CD indices were investigated through a literature search.

**Fig. 2:**
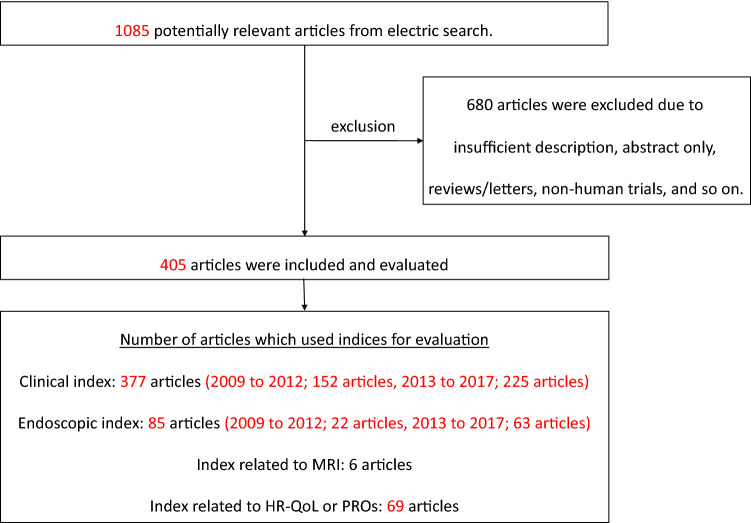
Flow diagram of the study selection process for CD. We performed an electronic search using PubMed. The survey period was from January 2009 to December 2017. The search string we used included “Crohn’s disease” and “clinical trial” as key words. We carefully read the studies that were extracted, and 405 studies were considered to have been evaluated by an index

We performed an electronic research using PubMed with a survey period spanning from January 2009 to December 2017. “Crohn’s disease” and “clinical trial” were included in the search string, and 405 articles that had been regarded as evaluated with CI, EI, index using magnetic resonance imaging (MRI), and index using patient-reported outcomes (PROs) or health-related quality of life (HR-QoL) were extracted and read through carefully. Figure 2 shows the flow diagram of the study selection process, including the exclusion criteria used. Tables 3, 4, 5, and 6 show the frequency of use of CI, EI, index related to MRI, and index related to PROs or HR-QoL based on these research results. The indices that were revealed to be used most frequently in previous studies, considered to be highly valid, and appeared to be frequently applicable in future, were selected, and their development reasons, evaluation items, characteristics, and various definitions were reviewed. Additionally, the index for pediatric CD and the index intended to evaluate small bowel lesions, such as indices related to capsule endoscopy (CE) or MRI, are described independently from the results of the research.

**Table 3:** Number and frequency of CIs in recent clinical trials for CD (*N* = 377, including duplicates)

Survey period	Clinical index	Validation	Number (%)
2009 to 2012 (*N* = 152, including duplicates)			
	Crohnʼs Disease Activity Index (CDAI)	Validated	125 (82.2%)
	Harvey–Bradshaw Index (Simple CDAI)	Validated	23 (15.1%)
	Pediatric Crohnʼs Disease Activity Index (PCDAI)	Validated	7 (4.6%)
	Perianal Crohnʼs Disease Activity Index	Validated	3 (2.0%)
	IOIBD Score (Oxford Score)	Validated	2 (1.3%)
	Others (Including original indices for the trial)	Not available	8 (5.3%)
2013–2017 (*N* = 225, including duplicates)			
	Crohnʼs Disease Activity Index (CDAI)	Validated	129 (57.3%)
	Harvey–Bradshaw Index (Simple CDAI)	Validated	38 (16.9%)
	Pediatric Crohnʼs Disease Activity Index (PCDAI)	Validated	35 (15.6%)
	IOIBD Score (Oxford Score)	Validated	2 (0.9%)
	Others (Including original indices for the trial)	Not available	16 (7.1%)
2009–2017 (*N* = 377, including duplicates)			
	Crohnʼs Disease Activity Index (CDAI)	Validated	254 (67.4%)
	Harvey–Bradshaw Index (Simple CDAI)	Validated	61 (16.2%)
	Pediatric Crohnʼs Disease Activity Index (PCDAI)	Validated	42 (11.1%)
	IOIBD Score (Oxford Score)	Validated	4 (1.1%)
	Perianal Crohnʼs Disease Activity Index	Validated	3 (0.8%)
	Others (Including original indices for the trial)	Not available	24 (6.4%)

*CIs* Clinical indices, *CD* Crohn’s disease, *IOIBD* International Organization for the Study of Inflammatory Bowel Disease

**Table 4:** Number and frequency of EIs in recent clinical trials for CD (*N* = 85, including duplicates)

Survey period	Endoscopic index	Validation	Number (%)
2009–2012 (*N* = 22, including duplicates)			
	Crohnʼs Disease Endoscopic Index of Severity (CDEIS)	Validated	10 (45.5)
	Rutgeerts Score	Validated	6 (27.3)
	Capsule Endoscopy Crohnʼs Disease Activity Index (CECDAI)	Validated	3 (13.6)
	Simple Endoscopic Score for Crohnʼs Disease (SES-CD)	Validated	2 (9.1)
	Others (Including original indices for the trial)	Not available	5 (7.0)
2013–2017 (*N* = 63, including duplicates)			
	Simple Endoscopic Score for Crohnʼs Disease (SES-CD)	Validated	31 (49.2)
	Rutgeerts Score	Validated	23 (36.5)
	Crohnʼs Disease Endoscopic Index of Severity (CDEIS)	Validated	19 (30.2)
	Lewis Score (Capsule Endoscopy Score)	Validated	2 (3.2)
	Capsule Endoscopy Crohnʼs Disease Activity Index (CECDAI)	Validated	1 (1.6)
	Others (Including original indices for the trial)	Not available	2 (3.2)
2009 to 2017 (*N* = 85, including duplicates)			
	Simple Endoscopic Score for Crohnʼs Disease (SES-CD)	Validated	33 (38.8)
	Crohnʼs Disease Endoscopic Index of Severity (CDEIS)	Validated	29 (34.1)
	Rutgeerts Score	Validated	29 (34.1)
	Capsule Endoscopy Crohnʼs Disease Activity Index (CECDAI)	Validated	4 (4.7)
	Lewis Score (Capsule Endoscopy Score)	Validated	2 (2.4)
	Others (Including original indices for the trial)	Not available	4 (4.7)

*EIs* endoscopic indices, *CD* Crohn’s disease

**Table 5:** Number and frequency of indices related to MRI in recent clinical trials for CD (*N* = 6, including duplicates)

Index related to MRI	Validation	Number (%)
Van Assche Index	Validated	2 (33.3)
Magnetic Resonance Index of Activity (MRIA) Score	Validated	1 (16.7)
MRI Enterography Global (MEGS) Score	Validated	1 (16.7)
Magnetic Resonance Enterocolonography (MREC) Score	Validated	1 (16.7)
Others (Including original indices for the trial)	Not available	1 (16.7)

*MRI* magnetic resonance imaging, *CD* Crohn’s disease

**Table 6:** Number and frequency of indices related to PROs or HR-QoL in recent clinical trials for CD (*N* = 69, including duplicates)

Index related to PROs/HR-QoL	Validation	Number (%)
Inflammatory Bowel Disease Questionnaire (IBDQ)	Validated	50 (72.5)
Short Form-36 Health Survey Questionnaire (SF-36)	Validated	7 (10.1)
Others (Including original indices for the trial)	Not available	22 (31.9)

*PROs* patient-reported outcomes, *HR-QoL* health-related quality of life, *CD* Crohn’s disease

### Results of electronic research for CD

In terms recent (2009–2017) of CIs, the Crohn’s Disease Activity Index (CDAI) (67.4%), Harvey-Bradshaw index (Simple CDAI) (16.2%), Pediatric CDAI (11.1%), and others, were the most adopted indices. In terms of EI, the Simple Endoscopic Score for Crohn’s disease (SES-CD) (38.8%), Crohn’s Disease Endoscopic Index of Severity (CDEIS) (34.1%), and Rutgeerts Score (34.1%) were the most adopted indices. In terms of indices related to CE, the CE Crohn’s Disease Activity Index (CECDAI) (4.7%) and the Lewis Score (Capsule Endoscopy Score) (2.4%) were adopted. In terms of indices related to MRI, the Van Assche Index, Magnetic Resonance Index of Activity (MaRIA) Score, MRI Enterography Global Score (MEGS), and Magnetic Resonance Enterocolonography (MREC) Score were adopted at almost the same frequency. In terms of indices related to PROs or HR-QoL, the Inflammatory Bowel Disease Questionnaire (IBDQ) (72.5%) and Short Form-36 Health Survey Questionnaire (SF-36) (10.1%) were adopted.

In the section on CIs, CDAI and the Harvey-Bradshaw Index (Simple CDAI) are described. In the section on EIs, SES-CD, CDEIS, and Rutgeerts scores are described. Additionally, the significance of PROs or HR-QoL was evaluated. As the indices for evaluating small bowel lesions, the CECDAI and Lewis scores were described as the indices related to CE. MaRIA and MREC were described as the indices related to MRI.

### Clinical Index for CD

#### Crohn’s Disease Activity Index (Supplementary Table 12)

This index was developed by the National Cooperative Crohn’s Disease Study (NCCDS) to evaluate the efficacy of placebo-controlled prednisone, sulfasalazine, and azathioprine on CD patients [62]. In 1976, eight variable items currently being applied then were selected from a study by multiple regression analysis [63]. In 1979, a re-evaluation of CDAI was performed [64]. Furthermore, a validation study of the selected eight variables was performed on cases attended by the NCCDS and TAS Study, a trial of sulfasalazine as adjunctive therapy for CD patients [65]. As a result, the similarity was shown in two multiple regression analyses, and it was clarified that the selected eight variable items were suitable for the evaluation of CD. For these reasons, the validity of the CDAI was rigorously verified and is currently positioned as the gold standard in CD evaluation, demonstrating its extremely high acceptance rate in clinical trials.

##### Problems included in CDAI and alternative indices

CDAI is a useful index, but it still has a number of problems [5, 61, 66–68]. (1) There are evaluation items that may vary from subject to subject, such as general condition and abdominal pain, which may affect reliability and validity. (2) The index cannot be calculated in the case of ileostomy or colostomy. (3) Retrospective evaluation may not accurately evaluate CD symptoms. (4) The standard body weight required by the evaluation was not clearly defined. (5) To unify the weighting of the scores among subjects, it is necessary to fully explain how to describe the scores; for example, the general condition is defined as good before the onset. (6) The severity of fistulas and stenosis cannot be accurately assessed by the index. (7) Finally, correlations between endoscopic activity and biomarkers are poor. Therefore, the following several indices are attracting attention as alternative indices of CDAI [67, 68].

#### Harvey-Bradshaw Index (Supplementary Table 13)

CDAI requires seven days to evaluate and is complicated because it consists of many items, including clinical tests. Harvey et al. developed this index to solve the complexity of CDAI [69]. The Harvey–Bradshaw Index (also known as Simple CDAI) consists of five evaluation items; three subjective clinical symptoms on the previous day, and two physical examination findings at the time of evaluation. Although it is easier to calculate than CDAI, it shows a high correlation with CDAI, and its acceptance rate in recent clinical trials is high. However, its problem is that its resulting score greatly depends on the number of liquid stools passed per day, so a CD patient with an increased number of liquid stools with intestinal resection or irritable bowel syndrome is likely to show a higher score that is disproportionate to the actual disease activity [70].

### Evaluation significance of patient-reported outcomes or health-related quality of life

PROs are attracting attention as alternatives to CDAI [67, 68]. PROs provide clues as to what the patient feels as an important problem without the burden of undergoing examinations, leading to improvement in the HR-QoL of the patient [71, 72]. Recently, along with CR, achievement of living remission, such as normalized HR-QoL or absence of disability, has come to be regarded as a therapeutic target, which various PROs have presented [70]. Table 6 summarizes 69 reports in which the index related to PROs or HR-QoL was used in a recent clinical trial of CD. Among them, the IBDQ [73] was most often adopted, which was followed by the SF-36 [74]. In cases wherein disease activity of CD is low, PROs may lead to be high if psychological disorder or irritable bowel syndrome coexist with CD. Therefore, it should be noted that there are some cases in which the score of the index related to PROs or HR-QoL does not necessarily reflect disease activity [17].

### Endoscopic Index for CD

#### Crohn’s Disease Endoscopic Index of Severity (Supplementary Table 14)

Mary et al. developed this index to standardize the endoscopic severity assessment of CD [75]. Based on endoscopic data, “global evaluation of lesion severity (GELS)” was set as dependent variable, and “average surface involved by the disease”, “average surface involved by ulcerations only”, “present, non-ulcerated, and ulcerated stenosis”, and “ number of segments exhibiting the lesion divided by the number of explored segments” were set as independent variables. Multiple regression analysis then determined six coefficients, including all six variables. The correlation between CDEIS and GELS was evaluated twice, and both showed a high correlation. Additionally, there was a high correlation observed between CDEIS and GELS for changes in patients who underwent endoscopy twice (first time: clinically active period; second time: 3–5 weeks after taking oral prednisone). Thus, CDEIS is a well-validated EI.

#### Simple Endoscopic Score for Crohn’s Disease (Supplementary Table 15)

CDEIS is an EI whose validity has been verified, but its calculation remains to be complicated and not necessarily easy to apply in daily clinical practice or clinical trials. Therefore, Daperno et al. developed SES-CD to create an EI that is easy to calculate and also highly valid [76]. The evaluation items of the CDEIS were referred to, and “size of ulcers”, “ulcerated surface”, “affected surface”, and “presence of narrowing” were selected as variables in SES-CD. Although it is a simpler index than is CDEIS, it is still not easy to use in daily clinical practice in terms of scoring complexity. Additionally, there are some reports pointing out difference between SES-CD and clinical disease activity [77–79].

#### Rutgeerts Score (Supplementary Table 16)

Rutgeert et al. developed and applied this index to confirm prognostic relapse factors in CD patients who underwent ileocecal resection [80]. The subjects were patients with CD who underwent ileocecal resection or right hemicolectomy and were evaluated up to 30 cm from the anastomotic site with endoscopy. The risk of re-surgery was evaluated by performing univariate analysis and regression analysis with the stepwise method for various patient backgrounds and endoscopic findings. The postoperative endoscopic findings and preoperative disease activity were shown to have a strong relationship statistically. Based on this result, they concluded that the endoscopic activity of the anastomotic site at one year after surgery most reflects the prognosis, and classified the endoscopic severity of the anastomotic site from i0 to i4.

### Index for anal lesions on CD

Although CDAI is a frequently used index, it cannot reflect symptoms caused by anal lesions, which have a great effect on HR-QoL in perforative CD [63, 64]. Therefore, an evaluation using an independent index for anal lesions of CD is desirable. Considering these, this section describes perianal CDAI.

#### Perianal Crohn’s Disease Activity Index (Supplementary Table 17)

Irvine et al. developed this index to independently and objectively evaluate anal lesions on CD [81]. The evaluation items consisted of five items, and in addition to the clinical evaluation items, HR-QoL was evaluated by the patient regarding pain and restriction of sexual activity. Losco et al. reported that the cutoff value of 4 maximizes the sensitivity and specificity of PDAI (sensitivity: 87%, specificity: 81%) [82].

### Index for evaluating small intestinal lesion of CD

Small intestinal lesions are frequently observed in patients with CD. However, it is not easy to evaluate small intestinal lesions, mainly for anatomical reasons. However, balloon-assisted enteroscopy (BAE) and capsule endoscopy (CE) have recently emerged as new endoscopic modalities and are used to evaluate the small intestinal mucosal severity of CD. Additionally, as shown in Table 5, recent clinical trials have also applied indices related to MRI, which can evaluate the transmural severity of CD. Each modality has its own advantages and disadvantages, and it is not always possible to evaluate the entire small intestine with a single modality. Available modalities are therefore recommended to be used complementarily when necessary. This section describes indices related to CE (Lewis Score, CECDAI) and indices related to MRI (MaRIA Score, MREC Score).

#### Lewis Score (Supplementary Table 18)

Gralnek et al. developed this index to score inflammatory changes seen in the small intestine based on CE findings [83]. The three findings of villous edema, ulcer, and stenosis, which showed a high concordance rate among observers, were defined as the evaluation items for inflammatory changes. The small intestine transit time of the CE is divided into three equal parts (1st–3rd tertile). Based on this division, the Lewis Score is the sum of the highest score, which is the sum of the villus appearance score and the ulcer score for each tertile, and the stenosis score for the entire small intestine. In the original article, < 135 was defined as the threshold to consider remission. However, it should be noted that this index is not specialized for CD, but for non-specific inflammatory findings found in other diseases.

#### Capsule Endoscopy Crohn’s Disease Activity Index (Supplementary Table 19)

Gal et al. developed this index to evaluate the activity of CDs using CE [84]. This index consists of three items: inflammation score, extent of disease score, and narrowing (stricture). Small bowel transit time (SBTT) was divided into two equal parts. CECDAI is a quantitative score that evaluates these three items and summarizes the scores. In the original article, the total scores of four physicians for the same patient showed similarity, and the correlation coefficient of Spearman was also high (0.8–0.93, *p* < 0.001). However, although CE itself is limited, capsule retention and incomplete observation of the entire small intestine (approximately 20%) have been identified. The definition of MH or endoscopic severity was not presented in the original article.

##### CECDAIic Score (Supplementary Table 20)

Compared to ileocolonoscopy, although a poorer completion rate due to insufficient bowel preparation and the risk of retention due to stricture were mentioned, colon CE is an expected alternative examination for ileocolonoscopy in the evaluation of the colonic mucosa [85]. In 2018, Niv et al. developed CECDAIic, in which the evaluation target site of CECDAI was “extended” to the colon [86].

#### Magnetic Resonance Index of Activity Score (Supplementary Table 21)

Rimola et al. developed this index to evaluate CD activity using MRI [87]. It was confirmed that the score showed a good correlation with HBI and CDEIS. Although the cutoff value and definition have not been clearly defined, if MH is defined as MRA score < 7, sensitivity and specificity are 85% and 78%, respectively [88]. Additionally, although the original study examined the large intestine and the terminal ileum only, sensitivity and specificity were observed at 87% and 86%, respectively, for the diagnostic ability of MH upon the application of the index [89]. In the original article, the definition was not clearly determined, but “MaRIA Score ≤ 7” was used as the definition of inactive CD [5].

#### Magnetic Resonance Enterocolonography Score (Supplementary Table 22)

Takenaka et al. developed this index to compare MRI and endoscopic findings [90]. Unlike the MaRIA score, the MREC score rates each finding and eliminates complicated calculations. The MREC Score showed a good correlation with SES-CD and modified Rutgeerts score, slightly weak correlation with CDAI, and no statistically significant correlation with CRP. Thus, this index is considered a better proxy of the endoscopic findings than is the disease activity. However, it has been pointed out that its ability to identify stenosis is inferior to that of BAE. The definition of transmural remission or activity of CD is not presented in the original article.

### Index for pediatric CD

Pediatric CD cases, compared to adult CD cases, have more extensive and severe lesions at diagnosis, show a higher frequency of progressive advances, and show higher proportions of small intestinal lesions, and growth disorder is observed in 10–40% of patients with pediatric CD at diagnosis [45, 46, 91]. Therefore, in the clinical practice for managing pediatric CD, it is necessary to consider growth disorders and the risk of aggravation in a short period of time in children compared to adults [91]. This section describes pediatric CDAI, which was developed to address these problems.

#### Pediatric Crohn’s Disease Activity Index (Supplementary Table 23)

Hyams et al. developed this index to match the characteristics of pediatric CD [92]. The concordance rate between observers was evaluated, and a good concordance rate (*r* = 0.81) was observed. It also showed a good correlation with PGA (*r* = 0.77) and the modified Harvey-Bradshaw Score (*r* = 0.86). The characteristic of this index is that it reflects growth factors, such as changes in height and weight, in addition to symptoms and laboratory findings. This index should be applied at the time of CD diagnosis, and recovery of growth disorders after diagnosis should be evaluated using a growth rate curve. Moreover, its correlation with SES-CD is poor to fair [5].

## Definitions of each index

This section defines the indices described in this review. Additionally, the definitions recommended in STRIDE-II are underlined.

### Clinical Index for UC

With the advent of various therapeutic agents, treatment of UC is advancing day by day; both treatment options and treatment diversity are increasing. In terms of determining therapeutic effectiveness for severe cases, indices that can reflect short-term response sharply tend to be applied more frequently. In contrast, evaluation of short-term response is not important for drugs, such as 5-ASA or immuno-modulators, and short-term fluctuations are not usually required for the index. In other words, the choice of CI and whether CR or clinical response is defined depends on the type of therapeutic agent being used as well as the characteristics of the clinical trial. Therefore, to utilize CI, its characteristics and precisely how to use it must be understood. Therefore, this section summarizes the definitions frequently used in clinical trials of each of the CIs outlined. The results are presented in Table 7.

**Table 7:** Definitions of CI for UC

Index	Definition
Mayo Score	
Remission	≤ 1 [93, 94], ≤ 2 [5, 20, 21, 44, 93, 95–97], ≤ 2 and no subscore > 1 and rectal bleeding score = 0 [98–106], < 2 [5, 20, 21, 107, 108], < 4 and rectal bleeding = 0 and endoscopy ≤ 1 [5, 20, 21], < 4 [5, 20, 21]
Response	≥ Δ3 [95], ≥ Δ2 and ≥ 25% decrease with Δrectal bleeding ≥ 1 or ≤ 1 [101], > Δ3 and at least a 30% decrease [97,99] < 3 and ≥ Δ30% with Δrectal bleeding < 1 with rectal bleeding ≤ 1 [44, 93, 96, 100, 102–106], rectal bleeding ≤ 1 [106]
Partial Mayo Score (p-MS)	
Remission	≤ 2 [5, 100, 106], < 2 [5], ≤ 2 with no subscore > 1 [98, 99], < 3 and no score > 1 [5]
Response	≥ 1 [99], ≥ Δ1 [99, 107], ≥ Δ2 [5, 20, 21, 99], ≥ Δ2 and ≥ Δ30% [100], > Δ2 [5, 20, 21], ≥ 2 and ≥ Δ25% with Δrectal bleeding subscore ≥ 1 or ≤ 1 [68], > Δ3 [5, 20, 21], ≥ Δ30% [5, 20, 21], ≥ Δ30% and Δeach sub-score ≥ 1 [5, 20, 21, 100]
Sutherland Index (also known as Disease Activity Index, DAI)	
Remission	= 0 [109–112], ≤ 1 [113, 114], ≤ 2 with no individual subscore > 1 [109, 115], ≤ 2 with subscore ≤ 1 [115], ≤ 3 [116]
Improvement	≥ Δ2 [110], ≤ Δ3 [112], ≥ Δ4 with improvement of all categorizes [109, 115]
Modified DAI	
Remission	= 0 [112]
Improvement	≤ Δ3 [112]
Rachmilewitz Index (also known as Clinical Activity Index, CAI)	
Remission	CAI ≤ 4 [24, 117–119]
Response	CAI ≥ Δ1 [119]
Improvement	CAI ≥ Δ3 [117, 118]
Simple Clinical Colitis Activity Index (SCCAI)	
Remission	≤ 2 [5, 99]
Response	Δ > 30% [5]
Lichtiger Index	
Remission	≤ 4 [120, 121]
Improvement	≤ 10 and ≥ Δ3 [120]
Pediatric Ulcerative Colitis Activity Index (PUCAI)	
Remission	≤ 10 points [5, 122]
Response	ΔPUCAI≧20 [5]

*CI* clinical index, *UC* ulcerative colitis

### Endoscopic Index for UC

In many clinical trials of UC, in particular, new drugs, ER or MH are listed as primary or secondary evaluation endpoints. However, its definition currently differs per clinical trial. For example, MH is defined as 0 or 1 in MES in many papers, but recently, it has been required to aim for MES 0 to improve the long-term clinical course [5, 13–15]. Additionally, the definition of MH by UCEIS, whose validity has been verified and proposed, is increasingly being adopted. Therefore, this section summarizes the definitions frequently used in clinical trials for each of the outlined EIs. The results are presented in Table 8.

**Table 8:** Definitions of EI for UC

Index	Definition
Mayo Endoscopic Sub-score (MES)	
Remission (mucosal healing)	= 0 [5, 20, 21, 43, 94, 103, 108, 116], ≤ 1 [5, 20, 21, 43, 44, 93, 96–102, 104–107, 120, 121, 123, 124]
Response	≥ 1 point decrease [5, 20, 21, 103]
Ulcerative Colitis Endoscopic Index of Severity (UCEIS)	
Remission	= 0 [5, 21], ≤ 1 [5, 21]
Response	ΔUCEIS ≥ 2 [5, 21]
Sutherland Index (also known as Disease Activity Index, DAI)	
Endoscopic remission	EI ≤ 1 [109–111, 115, 125]
Endoscopic improvement	Decrease in EI [110]
Modified DAI	
Remission	EI < 1 [111], EI ≤ 1 [113], normal mucosal appearance [114]
Rachmilewitz Index (also known as Clinical Activity Index, CAI)	
Endoscopic remission	EI < 4 [117, 118], EI ≤ 4 [119]
Mucosal healing	EI = 0 [119]
Improvement	EI ≥ Δ1 [117, 118]

*EI* endoscopic index, *UC* ulcerative colitis

### Clinical Index for CD

CDAI can be said to be the champion index of CI for CD, and has it been adopted in most clinical trials. The definition of CR was defined as CDAI < 150, which now appears to be the gold standard. However, definitions of response, relapse, and active are defined but used in various ways in different trial. Therefore, especially for CDAI, the results for these definitions are described in detail. Additionally, the disease characteristics of CD include many cases of onset at a young age with a high rate of anal lesions. Therefore, pediatric CDAI and perianal CDAI are highly needed as separate evaluation indices independent of CDAI. Therefore, this section summarizes the definitions frequently used in clinical trials of each of the CIs outlined. The results are presented in Table 9.

**Table 9:** Definitions of CI for CD

Index	Definition
Crohn’s Disease Activity Index (CDAI)	
Remission	< 150 [5, 69, 108, 126–136, 137, 138, 140], < 150 with ΔCDAI ≥ 100 [137, 138], < 175 [129], ≤ 200 [139]
Response	ΔCDAI 50 [140], ΔCDAI 70 [5, 126], ΔCDAI 75 [140], ΔCDAI 100 [5, 126–128, 130, 131, 140], < 150 [128, 130, 131]
Relapse	ΔCDAI > 100 [137], ≥ 150 [132, 133], > 150 [137], ≥ 150 with ΔCDAI ≥ 60 [133, 141], 150–250 with ΔCDAI > 50 [134], ≥ 150 with ΔCDAI ≥ 100 [142], > 200 [135], > 175 [129], ≥ 200 with ΔCDAI ≥ 60 [139, 143], > 220 with ΔCDAI ≥ 70 [126–128, 130], > 220 [128], > 250 [134, 143]
Active	ΔCDAI ≥ 100 [136], > 150 with ΔCDAI ≥ 100 [144], ≥ 200 [129, 132, 135, 137, 138, 140], > 200 with ΔCDAI ≥ 60 [139] > 220 with ΔCDAI ≥ 100 [131]
Mild	150–220 [5]
Moderate–severe	220–450 [5, 128]
Harvey–Bradshaw Index (simple CDAI)	
Remission	< 4 [5], ≤ 4 [145], < 5 [5]
Response	ΔHBI ≥ 3 [5], < 5 [1], ≥ Δ30% [5], ≥ Δ50% [5]
Active	> 4 [145], ≥ 7 [145]
Perianal Crohn’s Disease Activity Index (Perianal CDAI)	
Remission	= 0 [82]
Pediatric Crohn’s Disease Activity Index (Pediatric CDAI)	
Remission	< 7.5 excluding the height item [5], < 10 [5]
Response	ΔPCDAI ≥ 12.5 [5]
Weighting Pediatric Crohn’s Disease Activity Index (wPCDAI)	
Remission	< 12.5 [5]
Response	ΔwPCDAI ≥ 17.5 [5]

*CI* clinical index, *CD* Crohn’s disease

### Endoscopic Index for CD

In CD, an evaluation method for colorectal lesions has been established, but there remains to be no consensus on the evaluation of small intestinal lesions. Definitions of MH and transluminal healing using CT and MRI have also been proposed [6]. Considering these, this section summarizes the definition of ER or MH used in clinical trials to better understand the above trends and issues. The results are presented in Table 10.

**Table 10:** Definitions of EI for CD

Index	Definition
Crohn’s Disease Endoscopic Index of Severity (CDEIS)	
Remission	No ulcer [5, 127], ≤ 2 with isolated ileitis [127,128] < 3 [5], < 4 [77], ≤ 4 [73, 126, 127], ≤ 4 for patients with isolated ileitis, < 6 [5, 128]
Mucosal healing	absence of mucosal ulceration [5, 108], < 3 [5]
Response	> 50% decrease [5, 127, 128]
Simple Endoscopic Score for Crohn’s Disease (SES-CD)	
Remission	Ulcer sub-score = 0 (including aphthous ulcerations) [5], < 1 [5], < 2 [102], < 3 [5, 108], < 4 [5], ≦4 and ΔSES-CD≧2 [108], < 5 [5], < 6 [5]
Response	> 50% decrease [5]
Rutgeerts Score	
Remission	≤ 2 [24]

*EI* endoscopic index, *CD* Crohn’s disease

#### Summary and conclusion

This survey clarified the use of CIs and EIs in recent clinical trials for IBD. The index related to HR-QoL or PROs and the index using new modalities, such as CE and MRI, were used in certain frequencies depending on the application. Based on the survey results, we outlined the main indices deemed to be most useful in clinical practice and clinical trial planning. Additionally, the definition of CR and MH was explained as much as possible. Recently, the treatment of IBD has progressed, and along with this, methods of evaluating its activity based on treatment targets and long-term perspectives have also changed. At present, MH remains to be a valid therapeutic target, and endoscopic evaluation also remains to be emphasized in managing IBD. However, frequent monitoring using invasive endoscopy is not feasible. Therefore, indices and biomarkers that correlate with endoscopic severity were investigated. Alternatively, as suggested by STRIDE-II, the evaluation method based on the concept of T2T, which achieves the treatment target according to the treatment timing, is being questioned. In other words, the targets are improvement of symptoms and PRO in the short term, normalization of biomarkers in the intermediate term, and ER and HR in the long term. Corresponding indices and biomarkers were selected for each target. It is important for physicians engaged in managing IBD to know the characteristics of each index and biomarker, as well as to select and utilize the appropriate index and biomarker that match their treatment targets in clinical settings or clinical trials.

## Supplementary Information

Below is the link to the electronic supplementary material.

Supplementary file1 (DOCX 37 KB)

## Abbreviations

IBD: Inflammatory bowel disease
CI: Clinical index
EI: Endoscopic index
MH: Mucosal healing
PH: Pathological healing
T2T: Treat to target
CRP: C-reactive protein
FC: Fecal calprotectin
UC: Ulcerative colitis
CD: Crohn’s disease
DAI: Disease activity index
CAI: Clinical activity index
SCCAI: Simple Clinical Colitis Activity Index
MES: Mayo Endoscopic Sub-score
UCEIS: Ulcerative Colitis Endoscopic Index of Severity
CR: Complete response
PR: Partial response
UCCS: Ulcerative Colitis Clinical Score
PGA: Physician’s Global Assessment
p-MS: Partial Mayo Score
ER: Endoscopic remission
PUCAI: Pediatric UC Activity Index
PDAI: Pouchitis Disease Activity Index
MRI: Magnetic resonance imaging
PROs: Patient-reported outcomes
HR-QoL: Health-related quality of life
CE: Capsule endoscopy
CDAI: Crohn’s Disease Activity Index
SES-CD: Simple Endoscopic Score for CD
CDEIS: Crohn’s Disease Endoscopic Index of Severity
CECDAI: CE Crohn’s Disease Activity Index
MaRIA: Magnetic Resonance Index of Activity
MEGS: MRI Enterography Global Score
MREC: Magnetic resonance enterocolonography
IBDQ: Inflammatory Bowel Disease Questionnaire
SF-36: Short Form-36 Health Survey Questionnaire
NCCDS: National Cooperative Crohn’s Disease Study
GELS: Global evaluation of lesion severity
BAE: Balloon-assisted enteroscopy
CE: Capsule endoscopy
