# Supplementary material for: A review on the current status and definitions of activity indices in inflammatory bowel disease: how to use indices for precise evaluation
Source: J Gastroenterol. 2022 Mar 2;57(4):246–66. doi: 10.1007/s00535-022-01862-y (PMC8938394; doi:10.1007/s00535-022-01862-y)
Supplement: Supplementary file 1 — Supplementary file1 (DOCX 37 KB) [file 535_2022_1862_MOESM1_ESM.docx]

Supplemental table 1.

| **Mayo score** |
| --- |
| **Stool frequency** |
| 0 = Normal number of stools for this patient  1 = 1-2 stools more than normal  2 = 3-4 stools more than normal  3 = 5 or more stools more than normal |
| **Rectal bleeding** |
| 0 = No blood seen  1 = Streaks or blood with stool less than one-half of the time  2 = Obvious blood with stool most of the time  3 = Blood alone passes |
| **Findings on endoscopy** |
| 0 = Normal or inactive disease  1 = Mild disease (erythema, decreased vascular pattern, and mild friability)  2 = Moderate disease (marked erythema, lack of vascular pattern, friability, and erosions)  3 = Severe disease (spontaneous bleeding and ulcerations) |
| **Physician’s global assessment** |
| 0 = Normal  1 = Mild disease  2 = Moderate disease  3 = Severe disease |

| **Sutherland index (also known as disease activity index, DAI)** |
| --- |
| **Stool frequency** |
| 0 = Normal  1 = 1-2 stools more than normal  2 = 3-4 stools more than normal  3 = 5 or more stools more than normal |
| **Rectal bleeding** |
| 0 = None  1 = Streaks of blood  2 = Obvious blood  3 = Mostly blood |
| **Mucosal appearance** |
| 0 = Normal  1 = Mild friability  2 = Moderate friability  3 = Exudation, spontaneous bleeding |
| **Physician’s rating of disease activity** |
| 0 = Normal  1 = Mild  2 = Moderate  3 = Severe |

Supplemental table 2.

Supplemental table 3.

| **Rachmilewitz index (also known as Clinical activity score, CAI)** |
| --- |
| **Clinical activity index** |
| **No of stools weekly** |
| 0 = <18  1 = 18-35  2 = 36-60  3 = >60 |
| **Blood in stools (based on weekly average)** |
| 0 = None  2 = Little  4 = A lot |
| **Investigator’s global assessment of symptomatic state** |
| 0 = Good  1 = Average  2 = Poor  3 = Very poor |
| **Abdominal pain/cramps** |
| 0 = None  1 = Mild  2 = Moderate  3 = Severe |
| **Temperature due to colitis (℃)** |
| 0 = 37-38  3 = >38 |
| **Extraintestinal manifestations** |
| 3 = Iritis  3 = Erythema nodosum  3 = Arthritis |
| **Lavoratory findings** |
| 1 = Sedimentation rate > 50mm in 1st h  2 = Sedimentation rate > 100mm in 1st h  4 = Hemoglobin <100 g/l |
| **Endoscopic index** |
| **Granulation scattering reflected light** |
| 0 = No  2 = Yes |
| **Vascular pattern** |
| 0 = Normal  1 = Faded/disturbed  2 = Completely absent |
| **Vulnerability of mucosa** |
| 0 = None  2 = Slightly increased (contact bleeding)  4 = Greatly increased (spontaneous bleeding) |
| **Mucosal damage (mucus, fibrin, exudate, erosions, ulcer)** |
| 0 = None  2 = Slight  4 = Pronounced |

Supplemental table 4.

| **Simple clinical colitis activity index (SCCAI)** |
| --- |
| **Bowel frequency (day)** |
| 0 = 1-3  1 = 4-6  2 = 7-9  3 = >9 |
| **Bowel frequency (night)** |
| 1 = 1-3  2 = 4-6 |
| **Urgency of defecation** |
| 1 = Hurry  2 = Immediately  3 = Incontinence |
| **Blood in stool** |
| 1 = Trace  2 = Occasionally frank  3 = Usually frank |
| **General well being** |
| 0 = Very well  1 = Slightly below par  2 = Poor  3 = Very poor  4 = Terrible |
| **Extracolonic features** |
| 1 per manifestation |

Supplemental table 5.

| **Lichtiger index** |
| --- |
| **Diarrhea (no. of daily tools)** |
| 0 = 0-2  1 = 3 or 4  2 = 5 or 6  3 = 7-9  4 = 10 |
| **Nocturnal diarrhea** |
| 0 = No  1 = Yes |
| **Visible blood in stool (% of movement)** |
| 0 = 0  1 = <50  2 = ≥50  3 = 100 |
| **Fecal incontinence** |
| 0 = No  1 = Yes |
| **Abdominal pain or cramping** |
| 0 = None  1 = Mild  2 = Moderate  3 = Severe |
| **General well being** |
| 0 = Perfect  1 = Very good  2 = Good  3 = Average  4 = Poor  5 = Terrible |
| **Abdominal tenderness** |
| 0 = None  1 = Mild and localized  2 = Mild to moderate and diffuse  3 = Severe or rebound |
| **Need for antidiarrheal drugs** |
| 0 = No  1 = Yes |

Supplemental table 6.

| **Ulcerative colitis endoscopic index of severity (UCEIS)** |
| --- |
| **Vascular pattern** |
| 1 = Normal  2 = Patchy obliteration  3 = Obliterated |
| **Bleeding** |
| 1 = None  2 = Mucosal  3 = Luminal mild  4 = Luminal moderate or severe |
| **Erosions and ulcers** |
| 1 = None  2 = Erosions  3 = Superficial ulcer  4 = Deep ulcer |

Supplemental table 7.

| **Matts classification** |
| --- |
| **Endoscopic grades** |
| Grade 1 = Normal  Grade 2 = Mild granularity of the mucosa with mild contact bleeding  Grade 3 = marked granularity and edema of the mucosa, contact bleeding, and spontaneous bleeding  Grade 4 = Severe ulceration of mucosa with hemorrhage |
| **Histologic grades** |
| Grade 1 = Normal appearance  Grade 2 = Some infiltration of the mucosa or lamina propria with either round cells or polymorphs  Grade 3 = Much cellular infiltration of the mucosa, lamina propria, and submucosa  Grade 4 = Presence of crypt abscesses, with much infiltration of all layers of the mucosa  Grade 5 = Ulceration, erosion, or necrosis of the mucosa, with cellular infiltration of some or all of its layers |

Supplemental table 8.

| **Riley score** |
| --- |
| **Acute inflammatory cell infiltrate (polymorphonuclear cells in the lamina propria)** |
| **Crypt abscesses** |
| **Mucin depletion** |
| **Surface epithelial integrity** |
| **Chronic inflammatory cell infiltrate (round cells in the lamina propria)** |
| **Crypt architectural irregularities** |
| Each feature was graded on a four point scale corresponding to none, mild, moderate, or severe. |

Supplemental table 9.

| **Geboes score** |
| --- |
| **Grade 0 Structural (architectural change)** |
| 0.0 No abnormality  0.1 Mild abnormality  0.2 Mild or moderate diffuse or multifocal abnormalities  0.3 Severe diffuse or multifocal abnormalities |
| **Grade 1 Chronic inflammatory infiltrate** |
| 1.0 No increase  1.1 Mild but unequivocal increase  1.2 Moderate increase  1.3 Marked increase |
| **Grade 2 Lamina propria neutrophils and eosinophils** |
| 2A Eosinophils |
| 2A. 0 No increase  2A.1 Mild but unequivocal increase  2A.2 Moderate increase  2A.3 Marked increase |
| 2B Neutrophils |
| 2B. 0 None  2B.1 Mild but unequivocal increase  2B.2 Moderate increase  2B.3 Marked increase |
| **Grade 3 Neutrophils in epithelium** |
| 3.0 None  3.1 < 5% crypts involved  3.2 < 50% crypts involved  3.3 > 50% crypts involved |
| **Grade 4 Crypt destruction** |
| 4.0 None  4.1 Probable—local excess of neutrophils in part of crypt  4.2 Probable—marked attenuation  4.3 Unequivocal crypt destruction |
| **Grade 5 Erosion or ulceration** |
| 5.0 No erosion, ulceration, or granulation tissue  5.1 Recovering epithelium+adjacent inflammation  5.2 Probable erosion—focally stripped  5.3 Unequivocal erosion  5.4 Ulcer or granulation tissue |

Supplemental table 10.

| **Pediatric ulcerative colitis activity index (PUCAI)** |
| --- |
| **Abdominal pain** |
| 0 = No pain  5 = Pain can be ignored  10 = Pain cannot be ignored |
| **Rectal bleeding** |
| 0 = None  10 = Small amount only, in less than 50% of stools  20 = Small amount with most stools  30 = Large amount (.50% of the stool content) |
| **Stool consistency of most stools** |
| 0 = No pain  5 = Pain can be ignored  10 = Pain cannot be ignored |
| **Abdominal pain** |
| 0 = Formed  5 = Partially formed  10 = Completely unformed |
| **Number of stools per 24 h** |
| 0 = 0-2  5 = 3-5  10 = 6-8  15 = >8 |
| **Nocturnal stools (any episode causing wakening)** |
| 0 = No  10 = Yes |
| **Activity level** |
| 0 = No limitation of activity  5 = Occasional limitation of activity  10 = Severe restricted activity |
| **SUM OF PUCAI (0–85)** |

Supplemental table 11.

| **Pouchitis disease activity index (PDAI)** |
| --- |
| **Clinical** |
| **Stool frequency** |
| 0 = Usual postoperative stool frequency  1 = 1–2 stools/day > postoperative usual  2 = 3 or more stools/day > postoperative usual |
| **Rectal bleeding** |
| 0 = None or rare  1 = Present daily |
| **Fecal urgency or abdominal cramps** |
| 0 = None  1 = Occasional  2 = Usual |
| **Fever (temperature > 37.8℃)** |
| 0 = Absent  1 = Present |
| **Endoscopic inflammation** |
| **Stool frequency** |
| 1 = Edema  1 = Granularity  1 = Friability  1 = Loss of vascular pattern  1 = Mucous exudates  1 = Ulceration |
| **Acute histologic inflammation** |
| **Polymorphic nuclear leukocyte infiltration** |
| 1 = Mild  2 = Moderate + crypt abscess  3 = Severe + crypt abscess |
| **Ulceration per low-power field (mean)** |
| 1 = <25%  2 = 25–50%  3 = >50% |

Supplemental table 12.

| **Crohn’s disease activity index (CDAI)** | |
| --- | --- |
| **Clinical or laboratory variables** | **Weighting factor** |
| **Number of liquid or soft stools each day for 7 days** | ×2 |
| **Abdominal pain each day for 7 days** | ×5 |
| 0=None  1=Mild,  2=Moderate  3=Severe |  |
| **General well being** | ×7 |
| 0=Well  1=Slightly under par  2=Poor  3=Very poor  4=Terrible) |  |
| **Number of complications** | ×20 |
| arthralgias, iritis, erythema nodosum, pyoderma gangrenosa, aphthous.ulcerations, anal fisuture, anal fistula, anal abscess, fever >37℃ past week, intestinal obstruction |  |
| **Opiates for diarrhea** | ×30 |
| 0=No  1=Yes |  |
| **Abdominal mass** | ×10 |
| 0=No  2=questionable  5=Yes |  |
| **Deviation from normal hematocrit** | ×6 |
| Male; 47  Female; 42 |  |
| **% deviation from standard weight** | ×1 |
| **Total CDAI** | |

Supplemental table 13.

| **Harvey-Bradshaw index (simple CDAI)** |
| --- |
| **General well being** |
| 0 = very well  1 = Slightly below par  2 = poor  3 = Very poor  4 = Terrible |
| **Abdominal pain** |
| 0 = None  1 = Mild  2 = Moderate  3 = Severe |
| **Number of liquid stools per day** |
| **Abdominal mass** |
| 0 = None  1 = Dubious  2 = Definite  3 = Difinite and tender |
| **Complications** |
| Arthralgia, uveitis, erythema nodosum, aphthous ulcers, pyoderma gangrenosum, anal fissure, new fistula, abscess (score 1 per item) |

Supplemental table 14.

| **Crohn’s disease endoscopic index of severity (CDEIS)** |
| --- |
| **CDEIS = 12×ISRCF (deep ulcerations)**  **+6×ISRCF (superficial ulcerations)**  **+ASSD**  **+ASSU**  **+3×PRES (non ulcerated stenosis)**  **+3×PRES (ulcerated stenosis)** |
| ISRCF: Number of segments exhibiting the lesion divided by the number of explored segments.  ASSD: Average surface involved by the disease.  ASSU: Average surface involved by ulcerations only.  PRES is taken to be 1 if the lesion is seen at least once at a given endoscopy and 0 otherwise. |

Supplemental table 15.

| **Simple endoscopic score for Crohn’s disease (SES-CD)** |
| --- |
| **Size of ulcers** |
| 0 = None  1 = Aphthous ulcers (φ0.1 to 0.5 cm)  2 = Large ulcers (φ0.5 to 2 cm)  3 = Very large ulcer (φ>2 cm) |
| **Ulcerated surface** |
| 0 = None  1 = <10%  2 = 10-30%  3 = >30% |
| **Affected surface** |
| 0 = Unaffected segment  1 = <50%  2 = 50-75%  3 =>75% |
| **Presence of narrowings** |
| 0 = None  1 = Single, can be passed  2 = Multiple, can be passed  3 = Cannnot be passed |
| **SES-CD = sum over all explored segments* (score for ulcers size + score for ulcerated surface + score for total affected surface + score for stenosis)**  * Ileum, right colon, transverse colon, left colon and rectum |

Supplemental table 16.

| **Rutgeerts score** |
| --- |
| i0 = no lesions  i1 = ≤ aphthous ulcers  i2 = >5 aphthous ulcers with normal intervening mucosa, skip area of larger lesions, or lesions confirmed to ileocolonic anastomosis  i3 = diffuse aphthous ileitis with diffusely inflamed mucosa  i4 = diffuse inflammation with larger ulcers, nodules and/or narrowing |

Supplemental table 17.

| **Perianal Crohn’s disease activity index (Perianal CDAI)** |
| --- |
| **Discharge** |
| 0 = No discharge  1 = Minimal mucous discharge  2 = Moderate mucous or purulent discharge  3 = Substantial discharge  4 = Gross fecal soiling |
| **Pain/restriction of activities** |
| 0 = No activity restriction  1 = Mild discomfort, no restriction  2 = Mod. discomfort, some limitation activities  3 = Marked discomfort, marked limitation  4 = Severe pain, severe limitation |
| **Restriction of sexual activity** |
| 0 = No restriction sexual activity  1 = Slight restriction sexual activity  2 = Mod. limitation sexual activity  3 = Marked limitation sexual activity  4 = Unable to engage in sexual activity |
| **Type of perianal disease** |
| 0 = No perianal disease/skin tags  1 = Anal fissure or mucosal tear  2 = <3 Perianal fistulae  3 = ≥3 Perianal fistulae  4 = Anal sphincter ulceration or fistulae with significant undermining of skin |
| **Degree of induration** |
| 0 = No induration  1 = Minimal induration  2 = Moderate induration  3 = Substantial induration  4 = Gross fluctuance/abscess |
| **Total score** |

Supplemental table 18.

| **Lewis score** | | | | | | |
| --- | --- | --- | --- | --- | --- | --- |
| **Parameters** | | **Number** | **Longitudinal extent** | | **Descriptors** |  |
| **First tertile** | | | | | | |
| Villous appearance | | 0 = Normal  1 = Oedematous | 8 = Short segment  12 = Long segment  20 = Whole tertile | | 1 = Single  14 = Patchy  17 = Diffuse |  |
| Ulcer | | 0 = None  3 = Single  5 = Few  10 = Multiple | 5 = Short segment  10 = Long segment  15 = Whole tertile | | 9 = <1/4  12 = 1/4-1/2  18 = >1/2 |  |
| **Second tertile** | | | | | | |
| Villous appearance | | 0 = Normal  1 = Oedematous | 8 = Short segment  12 = Long segment  20 = Whole tertile | | 1 = Single  14 = Patchy  17 = Diffuse |  |
| Ulcer | | 0 = None  3 = Single  5 = Few  10 = Multiple | 5 = Short segment  10 = Long segment  15 = Whole tertile | | 9 = <1/4  12 = 1/4-1/2  18 = >1/2 |  |
| **Third tertile** | | | | | | |
| Villous appearance | | 0 = Normal  1 = Oedematous | 8 = Short segment  12 = Long segment  20 = Whole tertile | | 1 = Single  14 = Patchy  17 = Diffuse |  |
| Ulcer | | 0 = None  3 = Single  5 = Few  10 = Multiple | 5 = Short segment  10 = Long segment  15 = Whole tertile | | 9 = <1/4  12 = 1/4-1/2  18 = >1/2 |  |
| **Stenosis – rated for whole study** | | | | | | |
| Stenosis | 0 = None  14 = Single  20 = Multiple | | | 24 = Ulcerated  2 = Non-ulcerated | 7 = Traversed  10 = Not traversed |  |
| **Lewis score = Maximum tertile score*+ (Stenosis number×ulcerated×traversed).**  *{[(Villous parameter×extent×descriptor) + (Ulcer parameter×extent×size)] for tertile 1 or [(Villous parameter×extent×descriptor) + (Ulcer parameter×extent×size)] for tertile 2 or [(Villous parameter×extent×descriptor) + (Ulcer parameter×extent×size)]for tertile 3} | | | | | | |

Supplemental table 19.

| **Capsule endoscopy Crohn’s disease activity index（CECDAI）** |
| --- |
| **A. Inflammation score** |
| 0 = None  1 = Mild to moderate edema/hyperemia/denudation  2 = Severe edema/hyperemia/denudation  3 = Bleeding, exudate, aphthous ulcer, erosion and small ulcer (<0.5 cm)  4 = Moderate ulcer (0.5–2 cm), pseudo polyp  5 = Large ulcer (>2 cm) |
| **B. Extent of disease score** |
| 0 = None  1 = Focal disease (single segment)  2 = Patchy disease (multiple segments)  3 = Diffuse disease |
| **C. Narrowing (stricture)** |
| 0 = None  1 = Single passed  2 = Multiple passed  3 = Obstruction |
| **Total Score = (A1×B1＋C1)＋(A2×B2＋C2).**  Segmental score = A×B＋C.  (1) Proximal small bowel; (2) Distal small bowel. |

**Supplemental table 20.**

| **Capsule endoscopy Crohn’s disease activity index（CECDAIic）** |
| --- |
| **A. Inflammation score** |
| 0 = None  1 = Mild to moderate edema/hyperemia/denudation  2 = Severe edema/hyperemia/denudation  3 = Bleeding, exudate, aphthous ulcer, erosion and small ulcer (<0.5 cm)  4 = Moderate ulcer (0.5–2 cm), pseudo polyp  5 = Large ulcer (>2 cm) |
| **B. Extent of disease score** |
| 0 = None  1 = Focal disease (single segment)  2 = Patchy disease (multiple segments)  3 = Diffuse disease |
| **C. Narrowing (stricture)** |
| 0 = None  1 = Single passed  2 = Multiple passed  3 = Obstruction |
| **Total Score = (A1×B1＋C1)＋(A2×B2＋C2)＋(A3×B3＋C3)＋(A4×B4＋C4).**  Segmental score = A×B＋C.  (1) Proximal small bowel; (2) Distal small bowel; (3) Right colon; (4) Left colon. |

Supplemental table **21**.

| **Magnetic resonance index of activity (MRIA) score** |
| --- |
| **MRIA (segment) = 1.5 ×wall thickness (mm)＋0.02 ×RCE*+5 ×oedema+10 ×ulceration**  *Relative contrast enhancement (RCE) =[(WSI postgadolinium-WSI pregadorinium)/(WSI pregadolinium))×100 (SD noise pregadolinium/SD noise postgadolinium), where SD noise pregadolinium corresponds to the average of three SDs of the signal intensity measured outside of the body before gadolinium injection, and SD noise postgadolinium corresponds to the SD of the same noise after gadolinium administration.] |

Supplemental table **22**.

| **Magnetic resonance enterocolonography（MREC）score** |
| --- |
| **Size of ulcers, wall thickness, highly enhancement, and deep depressions** |
| 0 = None  1 = Aphthousulcers (φ 0.1 to 0.5 cm)  2 = Largeulcers (φ 0.5 to 2 cm)  3 = Very large ulcers (φ> 2 cm) |
| **Ulcerated surface** |
| 0 = None  1 = <10%  2 = 10-30%  3 = >30% |
| **Affected surface when present hyperintensity on T2 relative to the signal of psoas muscle, and slightly enhancement on TI** |
| 0 = None  1 = <50 %  2 = 50-75 %  3 = >75 % |
| **Presence of narrowing** |
| 0 = >11 mm  1 = 11-6 mm  2 = 6mm>  3 = 6-0 mm |

Supplemental table **23**.

| **Pediatric Crohn’s disease activity index (Pediatric CDAI)** | | | |
| --- | --- | --- | --- |
| **HISTORY (Recall, 1 week)** | | | |
| **Abdominal pain** | | | |
| 0 = None  5 = Mild – Brief, does not interfere with activities  10 = Mod/severe daily, longer lasting, affects activities, nocturnal | | | |
| **Stools: per day** | | | |
| 0 = 0-1 liquid stools, no blood  5 = Up to 2 semi-formed with small blood, or 2-5 liquid  10 = Gross bleeding, or ≥ 6 liquid, or nocturnal diarrhea | | | |
| **Patient Functioning, General Well-Being (Recall, 1 week)** | | | |
| 0 = No limitation of activities, well  5 = Occasional difficulty in maintaining age appropriate activities  10 = Frequent limitation of activities, very poor | | | |
| **LABORATORY** | | | |
| **HCT (%)** | | | |
| <10 yrs | 11-14M | 15-19M | 11-19F |
| 0 = >33  2.5 = 28-32  5 = <28 | 0 = >35  2.5 = 30-34  5 = <30 | 0 = >37  2.5 = 32-36  5 = <32 | 0 = >34  2.5 = 29-33  5 = <29 |
| **ESR (mm/hr)** | | | |
| 0 = <20  2.5 = 20-50  5 = >50 | | | |
| **Albumin (g/dL)** | | | |
| 0 = ≥3.5  5 = 3.1-3.4  10 = ≤3.0 | | | |
| **EXAMINATION** | | | |
| **Weight** | | | |
| 0 = Weight gain or voluntary weight stable/loss  5 = Involuntary weight stable, weight loss 1-9 %  10 = Weight loss ≥ 10% | | | |
| **Height** | | | |
| **At Diagnosis** | | | |
| 0 = <1 channel decrease  5 = ≥1, <2 channel decrease  10 = ≥2 channel decrease | | | |
| **Follow-up** | | | |
| 0 = Height velocity ≥-1SD  5 = Height velocity <-1SD, >2SD  10 = Height velocity ≤-2SD | | | |
| **Abdomen** | | | |
| 0 = No tenderness, no mass  5 = Tenderness, or mass without tenderness  10 = Tenderness, involuntary guarding, definite mass | | | |
| **Perirectal disease** | | | |
| 0 = None, asymptomatic tags  5 = 1-2 indolent fistula, scant drainage, no tenderness  10 = Active fistula, drainage, tenderness, or bascess | | | |
| **Extra-intestinal Manifestations (Fever ≥38.5 for 3 days over past week, definite arthritis, uveitis, E.nodosum, P.gangrenosum)** | | | |
| 0 = None  5 = One  10 = ≥Two | | | |
| **TOTAL SCORE** | | | |
